# What are the neural networks that have been modulated by anatomically targeted therapies to promote recovery of consciousness? A scoping review

**DOI:** 10.3389/fneur.2026.1882395

**Published:** 2026-10-19

**Authors:** Roma D. Thakker, Mert Karabacak, Serhat Aydin, Bhargavi R. Budihal, Daniel D. Cummins, Fedor Panov, Konstantinos Margetis

**Affiliations:** 1 Department of Acute Medicine, London North West University Healthcare NHS Trust, London, United Kingdom; 2 Department of Neurosurgery, Mount Sinai Hospital, New York, NY, United States; 3 Department of Neurological Surgery, Koc Universitesi, İstanbul, Türkiye

**Keywords:** anatomical target, disorder of consciousness, neural network, neuromodulation, recovery

## Abstract

Disorders of Consciousness (DoC) arise from insults to neural networks responsible for awareness and arousal. DoC subtypes are frequently misdiagnosed, as mechanisms underlying consciousness remain poorly understood. Neurostimulation has been investigated as a potential therapy for DoC recovery; however, the evidence for its therapeutic efficacy remains heterogeneous, and the relationship between anatomical target, network modulation, and clinical recovery is unclear. This scoping review evaluates clinical trials investigating neurostimulation in DoC patients, observing trends and assessing the therapeutic significance of network-targeted stimulation, whilst proposing targets for future studies. A comprehensive scoping review was conducted in accordance with PRISMA extension for Scoping Reviews evaluating the efficacy of anatomically targeted neurostimulation in adult patients with DoC. Literature searches were conducted on PubMed, SCOPUS, and Web of Science, with the final search performed in March 2026. Studies were included if they reported quantitative measures of consciousness recovery and had a clearly defined stimulation target. Twenty-two studies were included. Thirteen evaluated the frontoparietal network, the with dorsolateral prefrontal cortex being the most frequent anatomical target, suggesting potential research bias toward this region. Transcranial direct current stimulation and repetitive transcranial magnetic stimulation were the most frequently trialed stimulation modalities. Among studies investigating electrophysiological measures or neuroimaging, improvements in functional connectivity were observed post-stimulation, often correlating with individual-level improvements in clinical consciousness. Nevertheless, group-level outcomes were inconsistent. Six studies reported improvement in consciousness-related behavioral outcomes. No single anatomical target was associated with a clinical response; however, heterogeneity amongst study cohorts and protocols confounded the ability to determine the relative efficacy of network targets. Network-targeted neurostimulation is a promising approach for DoC recovery but remains investigational. Current evidence does not support a single optimal anatomical target, with studies often overlooking individual variations in structural cerebral integrity and connectivity within their cohorts. Future trials should consider using individualized structural and functional mapping, stratify participants by baseline connectivity and clinical subtype, and apply standardized outcome definitions. A more personalized approach to neuromodulation will likely better optimize therapeutic outcomes for consciousness recovery.

## Introduction

Disorders of Consciousness (DoC) encompass a spectrum of clinical conditions characterized by insults to neural networks, resulting in prolonged states of altered wakefulness (1). DoC commonly results from traumatic brain injury (TBI), metabolic injury and anoxic insults (1). With a 37% misdiagnosis rate (2), the global incidence of DoC remains unknown with an approximation of annual incidence being 201 cases per 100,000 (3).

A diagnosis of DoC is multifaceted, requiring thorough clinical evaluation, electrophysiology testing and neuroimaging. DoC can be subdivided into four classifications: coma, vegetative state/unresponsive wakefulness syndrome (VS/UWS), minimally conscious state (MCS) and post-traumatic confusional state (4). MCS is characterized by partial preservation of consciousness and reflects a higher state of awareness compared to coma or VS/UWS (5). MCS is subdivided into MCS + (MCS with preservation of language), and MCS- (MCS without language) (4, 5). Diagnostic criteria that differentiate between these taxonomies rely on robust clinical behavioral assessment of the 2 key components of consciousness: awareness and arousal (6, 7). The Coma Recovery Scale-Revised (CRS-R) is the most utilized measure of clinical consciousness, with assessments such as the Glasgow Outcome Scale-Extended, Glasgow Coma Scale (GCS), and Disability Rating Scale, often used in conjunction (7).

The underlying mechanisms, and the complex interplay of the cerebral networks associated with DoC, remain poorly understood. Brain mapping has shown the default mode network (DMN) to be integral to consciousness (8). Responsible for internal awareness, it has been observed that DMN restoration in acute TBI patients in intensive care is positively correlated with consciousness recovery (9). Moreover, distinct topology patterns between the DMN and the Frontoparietal Network (FPN) are strong predictors of conscious state (10). The FPN is often referred to as the Central Executive Network (CEN) as this network aids executive function, attention and memory (11).

With the lack of formal, international therapeutic guidelines for DoC therapy, the American Academy of Neurology recommend that clinicians address confounding issues, perform serial assessments and focus on early neurorehabilitation. Interventional therapy for DoC includes pharmacological and neurostimulation approaches (7). Of the single pharmacological therapies, amantadine is the only agent supported by Level 1 evidence for significant acceleration in the rate of traumatic DoC recovery (12), with other medications including levodopa and zolpidem demonstrating positive effects at a lower-level of evidence (13). The efficacy of neurostimulation for DoC recovery remains inconsistent, as detailed in this review.

Across all DoC disorders, individuals with MCS are disproportionately represented in diagnostic and therapeutic trials (14). Whilst rates of misdiagnosis are higher amongst other DoC subclasses, the focus on MCS subjects is due to their relative neurological stability (15). These subjects are more likely to tolerate prolonged neurostimulatory interventions, including their potential adverse effects, allowing for more comprehensive evaluation of treatment efficacy (14). This scoping review aims to critically evaluate clinical trials which explore the efficacy of network specific neuromodulation in DoC subjects. This study will assess the clinical significance of the putative network modulation to guide current practice, whilst proposing efficacious targets for future studies.

## Methods

### Eligibility criteria

Studies included in this scoping review primarily assessed the efficacy of neurostimulatory therapies on DoC patients, with clearly defined anatomical targets for stimulation. Eligible studies included original peer-reviewed papers, written in English, with *n* > 1 participants. No publication timeline was set for article search given the slow evolution of neurostimulation therapy for DoC and limited high-level evidence over the years.

Studies were excluded if there was no quantitative measure of clinical consciousness recovery or if there was use of adjunct non-stimulatory treatments (e.g., pharmacological therapies) to minimize confounding. Studies without a control or comparator group were excluded. This restriction was applied as this scoping review aims to characterize whether there is an association between network-targeted stimulation and clinical DoC recovery. The existence of spontaneous recovery in DoC (particularly in traumatic etiologies (1)) confounds the ability of uncontrolled studies to differentiate between stimulation effects and natural recovery. Requiring a sham or control group as part of the inclusion criteria allows cross-study comparability of outcomes. It is acknowledged that this criterion may exclude lower-level evidence from this study that a scoping review would normally map.

Systematic reviews, meta-analyses, case reports, scoping reviews, commentaries and book chapters were excluded to ensure focus on primary research studies and reduce publication bias.

### Information sources and search

Advanced literature searches were conducted using PubMed, SCOPUS and Web of Science. Search strategy terms were combined with their Boolean operators with the full search strategy for PubMed is provided in the Supplementary material. The search strategy was developed and executed by the primary researcher and peer reviewed by an independent reviewer.

Records were exported to Covidence. An initial literature search was conducted on 11 September 2024. To ensure presentation of up-to-date evidence, a second literature search, using the same search strategy was performed on 22 March 2026, with any additional studies meeting the inclusion criteria incorporated into the final analysis. This scoping review was conducted and reported in accordance with the PRISMA extension for Scoping Reviews (PRISMA-ScR). The completed PRISMA-ScR checklist is provided in the Supplementary material.

### Data charting and items

Variables for data extraction were determined by two reviewers. Data charting was completed in Excel by the primary researcher following study screening. Extracted data included study characteristics (year, design), cohort characteristics (size, % females, mean age, DoC etiology, mean time since event), neurostimulation parameters (modality, duration of therapy, regime with accompanied sham periods, anatomical and neural network target), and measures of consciousness (tools used to measure consciousness recovery, including neurophysiology testing, imaging, and pre- & post- intervention measures of clinical consciousness).

### Synthesis of results

Studies were grouped descriptively according to anatomical stimulation site and putative neural network association; these categories were used to map the literature rather than to infer comparative effectiveness. Given that network nomenclature can vary across studies, assignments were matched using an anatomically informed framework. The FPN was defined by lateral prefrontal (including DLPFC) and posterior parietal regions; the DMN by medial prefrontal, posterior cingulate/precuneus, and lateral parietal regions; the sensorimotor network by pericentral motor and somatosensory regions; and thalamic/subcortical targets were classed separately from cortical functional networks. Classification for peripheral stimulation without a direct cortical target was based on its recognized mechanistic network in current literature. For instance, transauricular vagal nerve stimulation (taVNS) was categorized under the salience network based on the Vagal Cortical Pathways model (16). Studies stimulating anatomical sites spanning more than one canonical network were classified as cross-network interventions. The CRS-R is a reference measure for behavioral measure of consciousness as defined in prior DoC literature (1). Responders were defined by demonstrating a significant group-level improvement in CRS-R after active stimulation relative to sham or control (17). Studies where comparator groups were applied only to physiological or imaging outcomes, rather than clinical consciousness measures, the study was included but not included as demonstrating an observed controlled clinical improvement.

## Results

### Study characteristics

The initial search generated 1,318 studies, of which 539 were removed as duplicates. Titles and abstracts were screened by two researchers in accordance with the eligibility criteria. Thirty-six studies underwent a full-text review by the same individuals, and conflicts were resolved by a third independent reviewer. This resulted in nineteen studies which met the inclusion criteria.

To reflect the most recent evidence, an updated search using the same search strategy was conducted in March 2026. This identified 197 additional records. Following screening, 10 full-text studies were assessed with three studies meeting the inclusion criteria.

Twenty-two studies were included in the final analysis. Figure 1 summarizes both record searches, screening and inclusion.

**Figure 1:**
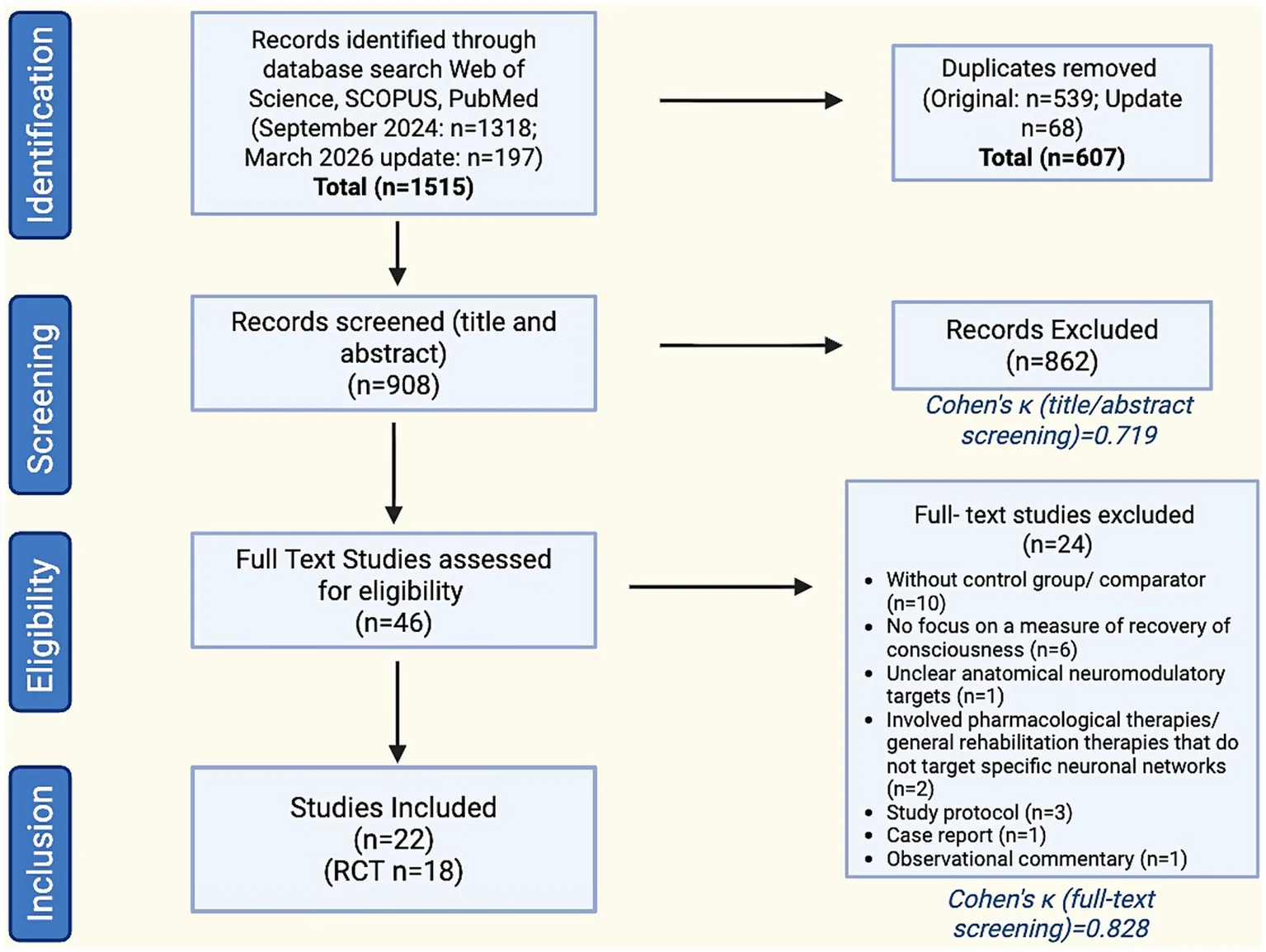
PRISMA flow diagram for literature search and inclusion. Cohen’s Kappa statistic specified (RCT, randomized control trial; PRISMA, Preferred Reporting Items for Systematic Review and Meta-Analyses). Figure created in BioRender. Thakker, R. (2026).

All studies were published between 2014 and 2026. The majority (*n* = 18) were randomized controlled trials (RCTs), with one retrospective cohort study. Whilst most employed sham controls, healthy controls (HCs) were used in 4 studies, with matched group characteristics (e.g., age, gender) to their respective counter DoC cohort. HCs underwent the same stimulation protocol as the DoC participants.

Table 1 provides a comparative overview of the anatomical targets and stimulation modalities used across all studies.

**Table 1:** Comparative overview of the putative networks and key anatomical regions targeted across studies meeting the search criteria (*N* = 22).

Putative network target	Network components	Primary target	Neuro-stimulation technique	Type of stimulation	DoC treated	DoC etiology	Study design (predominant)	Number of studies	Responders
Fronto-Parietal Network	DLPFC, posterior parietal cortex, frontal eye fields, DMPFC (partial), inferior parietal Lobule	DLPFC	tDCS, tPCS/tDCS rTMS, tACS, iTBS, tRNS	Non-invasive, peripheral	UWS, MCS, VS	TBI, anoxia, hemorrhage, stroke, cardiac arrest, infective (meningitis)	Randomized double-blind trial sham controlled, (cross-over; HC in subset)	13 12 single site (DLPFC) 1 multi-site	Thibaut et al. (21)
Frontoparietal Network and Default Mode Network	DLPFC and precuneus/posterior parietal cortex	DLPFC and parietal/precuneus	rTMS	Non-invasive	MCS	TBI and non-TBI	Randomized double-blind trial, sham-controlled (parallel)	1	Li et al. (27)
Default Mode Network	DMPFC, posterior cingulate cortex, precuneus, angular gyrus	Posterior parietal cortex/precuneus	rTMS	Non-invasive	MCS	TBI and non-TBI	Randomized double-blind trial sham controlled, (cross-over)	3	Xu et al. (24) Li et al. (25) Yan et al. (26)
Salience Network	Dorsal anterior cingulate, front-insular cortex, amygdala and temporal poles	Bilateral auricles	taVNS	Peripheral	MCS	TBI	Randomized double-blind trial sham controlled (parallel)	1	Zhou et al. (22)
Sensorimotor Network	M1, S1 cingulate cortex	M1	rTMS	Non-invasive	MCS, UWS, VS	TBI, hemorrhage, anoxia	Randomized double-blind trial sham controlled, (cross-over; HC in subset)	2	
Thalamo-cortical pathway	Thalamic nuclei; LGN, MGN, VPN and cortical regions, S1, M1, PFC, ACC, parietal cortex	Bilateral CM-Pf	DBS	Invasive	MCS	TBI, hemorrhage, anoxia	Randomized double-blind trial sham controlled, (cross-over)	1	
Motor Network	M1, basal ganglia, thalamus	Medial pallidum	DBS	Invasive	MCS, UWS	TBI, aneurysm, carotid aneurysm	Randomized double-blind trial sham controlled (cross-over)	1	

The neurostimulation technique, DoC cohort and quality, and frequency of study are identified. References: Fronto-parietal network (19–21, 23, 28, 34, 35, 44–49), Frontoparietal Network and default mode Network (27), default mode Network (24–26), salience Network (22), sensorimotor Network (36, 37), Thalamo-cortical pathway (38), motor Network (18).

DLFPC, dorsolateral prefrontal cortex; DMPFC, dorsomedial prefrontal cortex; tDCS, transcranial direct current stimulation; tPCS, transcranial pulsed current stimulation; rTMS, repetitive transcranial magnetic stimulation; tACS, transcranial alternating current stimulation; iTBS, intermittent theta burst stimulation; tRNS, transcranial random noise stimulation, UWS, unresponsive wakefulness syndrome; MCS, minimally conscious state; VS, vegetative state; TBI, traumatic brain injury; taVNS, transcutaneous auricular vagus nerve stimulation; M1, primary motor cortex; S1, primary somatosensory cortex; LGN, lateral geniculate nucleus; MGN, medial geniculate nucleus; VPN, ventral posterior nucleus; PFC, prefrontal cortex; ACC, anterior cingulate cortex; CM-Pf, Centromedian-Parafascicular nuclei; DBS, deep brain stimulation.

### Participant characteristics

All subjects were adults diagnosed with DoC of mixed etiologies. These ranged from TBI, anoxic injury, infection and hemorrhage. Several studies did not report specific DoC etiology, opting instead to label participants as “TBI” or “non-TBI.” Of those that specified, anoxic injury was the most frequent etiology, seen in cohorts of 11 studies. Whilst most studies focused on MCS patients, five studies had mixed DoC cohorts, including MCS, UWS and VS. Cohort sizes also varied, ranging between 3 and 50 individuals. Across study populations, mean age of participants was 48.1 years, with 43.7% females. Average time since injury or DoC causing event was 16.9 months.

### Stimulation modalities and targets

Repetitive transcranial magnetic stimulation (rTMS) and transcranial Direct Current Stimulation (tDCS) were the most frequently used stimulation modalities (both *n* = 8). Duration of stimulation ranged between 5 and 14 days, inclusive of the washout period. Longer stimulation durations were observed in a study utilizing deep brain stimulation (DBS) to the medial pallidum, extending up to 11 months (18).

The dorsolateral prefrontal cortex (DLPFC) was the most frequently targeted region (*n* = 14), reflecting a predominant focus on FPN modulation. Nevertheless, a wide range of anatomical stimulation sites were explored, with studies also targeting the primary motor cortex (M1), posterior parietal gyrus, medial pallidum and the bilateral Centromedian-Parafascicular (Cm-PF) nuclei. Several studies that targeted the DLPFC additionally engaged the broader FPN by simultaneously stimulating the posterior parietal cortices. For all studies, study level characteristics, targets and responder status are provided in Supplementary Table S1.

### Clinical consciousness/neurophysiological outcomes

All studies quantitatively measured consciousness with CRS-R, with 12 studies also using electroencephalogram (EEG) studies as a neurophysiological measure of recovery. Two studies opted for imaging including functional magnetic resonance imaging (fMRI) and fluorodeoxyglucose positron emission tomography. Alongside the CRS-R, one study simultaneously utilized the Western Neuro Sensory Stimulation Profile (WNSSP) neurobehavioral assessment tool (19). Four studies integrated additional functional outcome measures into their investigations, including the Glasgow Outcome Scale-Extended (GOSE) (20) (21), GCS (22), Disability Rating Scale (DRS) (22) and Clinical Global Impression-Improvement (CGI-I) (23). These tools differ from the CRS-R with regards to the parameters they assess and have limited utility for cross-study comparison.

Targeted neurostimulation was shown to improve neural connectivity (including increased alpha- band connectivity) which often translated to improvements in clinical consciousness and behavior, particularly at the individual-level. Only six of twenty-two studies reported significant improvements in clinical consciousness scores at group-level from baseline (CRS-R) following stimulation. Of these, one targeted the FPN (21) via the DLPFC, three targeted the DMN (24) (25) (26),via the posterior parietal cortex/precuneus, one targeted the FPN-DMN cross-network via combined DLPFC and parietal/precuneus stimulation (27) and one targeted the salience network (22). Notably, the FPN targeting study produced a transient significant positive improvement in CRS-R within the MCS sub-group (21). One further study targeting the FPN that reported significant CRS-R improvement was classed as a non-responder as this observation was not from a sham- comparison (28). There was no common stimulation modality between the six studies; four used rTMS, one used tDCS and one used taVNS. Cohort included both traumatic and non-traumatic etiologies with median time from inciting DoC event ranged approximately between 1.5 and 43 months. Five stimulation protocols used repeated multi-session stimulation protocols with one study using a single-session cross-over protocol (21). Among these six studies, there was no specific concentration of positive response at the most frequently studied network (the FPN).

### Safety and adverse events

Neurostimulation modalities generally demonstrated favorable safety profiles; however, the reporting of adverse events was inconsistent, with 11 studies not formally reporting any. Only one reported an adverse event secondary to their stimulation intervention (18). In this study, the treatment cohort received DBS to the medial pallidum. Whilst there was no perioperative mortality, one individual developed a bronchopulmonary infection. The patient received stimulation but passed away 15-months following electrode implantation, with no acute neurological signs prior to death. No other adverse events were reported. Participants who received transcutaneous auricular vagus nerve stimulation (taVNS) reported cases of seizures, pneumonitis and deep vein thrombosis in both treatment and sham-control groups (22). There was no significant difference in the frequency of these events between the groups, and they were not attributed to stimulation effects.

## Discussion

### Network role in consciousness

This scoping review demonstrates a predominant focus on the FPN, particularly by targeting the DLPFC in neurostimulation therapies for DoC. Established neuroimaging studies confirm the importance of thalamus-DLPFC tract structural integrity in maintaining clinical consciousness (29).

This focus may suggest that consciousness recovery can be driven by single network modulation. However, neural networks have many overlapping anatomical regions, and it is the complex interplay of these networks that give rise to consciousness (10, 30). For example, the studies classed in this review as engaging the DMN targeted the parietal hub via the posterior parietal cortex and precuneus. This likely co-modulated the FPN due to the shared parietal nodes between both networks (31, 32). Therefore, it is difficult to truly determine the significance of targeting an isolated anatomical region for DoC recovery. The positive outcomes observed across these studies may reflect wider coordinated restoration of neuronal activity across multiple networks as opposed to a single target (9, 10). The absence of a network-specific pattern for DoC recovery observed amongst these studies is not evidence that the chosen stimulation targets were irrelevant. Rather, the small number of responders and the heterogeneity amongst modality, DoC etiology and stimulation protocols limit the power of the dataset to conclude whether response was a network-specific effect. The network classifications applied in this review should be read as an organizing framework rather than evidence of selective network engagement.

Neurostimulation enables modulation of putative networks and its use, particularly for drug refractory neurological and psychiatric conditions, has increasing applications (33). The studies in this review explored various neuronal networks. Figure 2 depicts the functional profile and connections of these networks.

**Figure 2:**
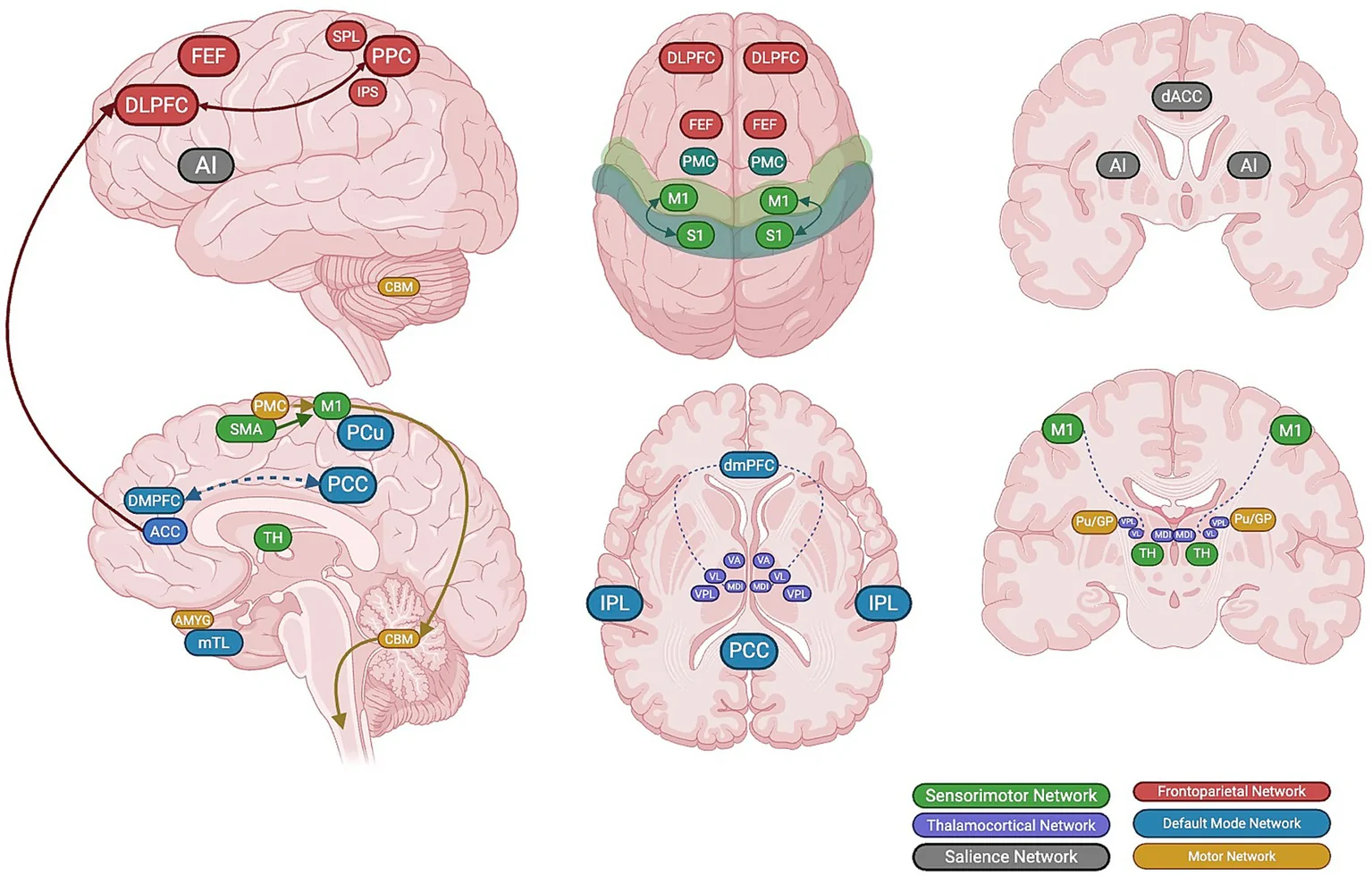
Functional profile of six neuronal networks integral for maintenance of consciousness, represented in the sagittal, axial and coronal planes (left to right). Key intra-network connections for each network are depicted: Sensorimotor Network- M1-S1 (sensory feedback for fine motor control), with PMC and SMA. Frontoparietal Network- DLPFC-PPC and FEF-DLPFC (executive control, top-down attention and memory). Default Mode Network- DMPFC-PPC and PCC-precuneus (internal awareness and mediating between task positive and negative tasks). Thalamocortical Network- Thalamic Relay VA, VL, VPL, MDI (arousal and sleep regulation). Motor Network- PMC/SMA-M1 (motor planning and execution). Salience Network- dACC and AI (mediates switch between DMN and FPN). (AI, anterior insula; ACC, anterior cingulate cortex; AMYG, amygdala; CBM, cerebellum; dACC, dorsal anterior cingulate cortex; DLPFC, dorsolateral prefrontal cortex; DMPFC, dorsomedial prefrontal cortex; FEF, frontal eye fields; FPN, fronto-parietal network; IPL, inferior parietal lobe; IPS, intraparietal sulcus; M1, primary motor cortex; MDI, medial dorsal nucleus; mTL, medial temporal lobe; PCC, posterior cingulate cortex; PCu, precuneus; PMC, premotor cortex; PPC, posterior parietal cortex; Pu/GP, putamen/globus pallidus; S1, primary sensory cortex; SMA, supplementary motor area; SPL, superior parietal lobe; TH, thalamus; VA, ventral anterior nucleus; VL, ventral lateral nucleus; VPL, ventral posterior nucleus). Figure created in BioRender. Thakker, R. (2026).

### Stimulation targets and clinical and neurophysiological outcomes

The FPN was targeted by several non-invasive stimulation modalities, most frequently tDCS and rTMS. This predominance is perhaps due to their earlier development and established clinical use compared to other non-invasive modalities like taVNS (33). Across all studies, both tDCS and rTMS maintained a good safety profile.

In studies employing dual-site stimulation, the DLPFC was frequently included as a target of choice. This approach enabled exploration of broader FPN modulation for consciousness recovery. For instance one study combined DLPFC and parietal/precuneus stimulation spanning both the FPN and DMN, whilst another chose to target frontoparietal regions F3/4 and CP5/6 bilaterally (27) (34). Clinical outcomes remained inconsistent, however, with the former demonstrating significant improvement in CRS-R, whilst the latter did not. These observations cannot establish advantage of multisite stimulation over single-target approaches.

Whilst several studies reported significant improvements in consciousness measures following DLPFC stimulation, findings were heterogenous. Many studies which employed the same modality of stimulation, with similar protocols to the DLPFC demonstrated no group-level improvement, with some cases of individual-level behavioral deterioration following intervention (34). It is likely that variability in stimulation protocol, DoC etiology and baseline structural and neural network integrity amongst cohorts influenced these findings (35).

Anatomical structural heterogeneity between DoC participants was frequently overlooked. For example, one study used EEG electrode landmarks to determine rTMS stimulation targets (24). Whilst practical, this approach is liable to human error secondary to individual anatomical variability, particularly in the context of potential structural brain alterations in DoC patients.

The other networks explored in this review also demonstrated variable therapeutic efficacy. Two studies targeting the sensorimotor network (SMN) via rTMS (36, 37), showed electrophysiological improvement and enhanced neural connectivity (though not statistically significant). Clinical improvements remained inconsistent, with one individual responding to sham stimulation. Both studies had brief stimulation durations (1-week before sham-switchover) which may suggest that shorter stimulation sessions are insufficient to bring about required neuroplastic changes for sustained consciousness recovery.

One study that observed the effects of 4-weeks taVNS on a mixed DoC cohort, targeting the Salience Network demonstrated significant improvements in functional consciousness recovery (22). These improvements were also sustained at 12 weeks and GCS significantly improved only in those receiving active stimulation.

DBS was used by two studies in this review. One targeted the thalamocortical pathway via the bilateral Centromedian-Parafascicular (Cm-Pf) nuclei complexes and found sustained improvement in clinical consciousness in three individuals, particularly in visual tracking skills, object recognition tasks and overall motor function (38). All subjects in this study, however, were individuals with stable MCS secondary to TBI. The distinction between long-term therapeutic effects of DBS and spontaneous recovery is unclear, particularly in a post-TBI population (39). As determined by Whishaw et al. spontaneous recovery following cerebral injury can be due to behavioral compensation, which in particular, will contribute to bias in long term follow up (40).

### Limitations and interpretation

Several key limitations were observed. Primarily, most studies included small cohorts limiting statistical power and generalizability of results across DoC populations. Furthermore, several studies trialed stimulation of mixed DoC populations, whilst others had MCS-only cohorts introducing substantial variability in baseline connectivity. This inconsistency in neural stability contributes to dilution of observable therapeutic group effects.

Although baseline connectivity and clinical consciousness were quantitatively measured prior to intervention, DoC subjects would often have heterogeneous etiologies, occurring over various timeframes, further confounding the ability to measure true potential of neurostimulation for long-term improvement. It is well established that despite its focal nature, TBI-related DoC can have prolonged recovery versus DoC secondary to anoxia, as strokes follow a more predictable pattern of injury allowing for better prognostication with targeted therapies (1). Moreover, DoC patients within the subacute phase following insult are more likely to exhibit spontaneous recovery which can falsely amplify the efficacy of neurostimulation relative to those DoC patients within the chronic phase, where endogenous improvement is minimal (1, 39).

The brief duration of stimulation, limited period of prospective follow-up and lack of dose–response data further confounds the ability to identify the true effects of neurostimulation efficacy and to determine optimal stimulation parameters. Cerebral recovery is optimized by sustained and repetitive periods of stimulation, through lasting synaptic reorganization (41), as demonstrated in patients with Parkinson’s Disease and stroke (42, 43) These limitations can increase publication bias and may overestimate the efficacy of neurostimulatory therapies for DoC.

Though this scoping review presents a relatively broad overview of studies, they are limited by their nature and may only provide superficial insight without establishing formal conclusions of treatment efficacy. The exclusion of studies without controls or comparators limits this review’s ability to capture the full breadth of existing evidence. Without establishing risk of bias, it is difficult to identify the true effect size of small study cohorts, which is a limitation of the scoping review methodology itself. Nevertheless, 18 studies were RCTs utilizing sham or healthy controls, increasing the overall reliability of this review’s findings.

### Future directions

Despite heterogeneous DoC etiologies, stimulation protocols were applied uniformly between subjects, neglecting critical individual variations in structural integrity and functional connectivity (which can be identified through fMRI or electrophysiology testing). Whilst these studies suggest potential positive therapeutic improvement in consciousness following anatomically targeted neurostimulation, they lacked neural connectivity-based stratification when formulating stimulation protocols.

Precise network stimulation through individualized mapping, guided by residual integrity (as opposed to anatomical landmarks) remains largely unexplored. Additionally, whilst dual-site stimulation was seen, targets were of the same network. The potential of dual-network stimulation or dual-modality stimulation has not yet been investigated. A shift toward tailored, patient-specific neuromodulation strategies may optimize the therapeutic applications of stimulation in DoC cohorts and move the field toward precision neurorehabilitation.

## Conclusion

Network-targeted neurostimulation appears feasible and generally safe across small, controlled studies of DoC populations. Several studies report individual-level improvements in CRS-R scores or neurophysiological connectivity. However, sustained group-level clinical benefit remains inconsistent, and current evidence does not identify a single optimal anatomical target. Whilst the DLPFC remains a promising target, its disproportionate evaluation relative to other networks reflects a research bias toward the FPN and potential limitation in the current research paradigm. Baseline connectivity remains a critical determinant of therapeutic efficacy which may restrict applications of therapeutic stimulation in patients with severe DoC. There is broad scope for future exploration, including combination stimulatory techniques, multi-network targeting, and using targeted stimulation in concordance with adjunct imaging and neurophysiological evaluation. Advancing research toward personalized neuromodulation will enhance diagnostic precision and therapeutic potential for DoC recovery.
