# Supplementary material for: What are the neural networks that have been modulated by anatomically targeted therapies to promote recovery of consciousness? A scoping review
Source: Front Neurol. 2026 Oct 19;17:1882395. doi: 10.3389/fneur.2026.1882395 (PMC13725107; doi:10.3389/fneur.2026.1882395)
Supplement: Supplementary file 1 [file Data_Sheet_1.pdf]

Preferred Reporting Items for Systematic reviews and Meta-Analyses extension for Scoping Reviews (PRISMA-ScR) Checklist

SECTION	ITEM	PRISMA-ScR CHECKLIST ITEM	REPORTED ON PAGE #
**TITLE**			
Title	1	Identify the report as a scoping review.	
**ABSTRACT**			
Structured summary	2	Provide a structured summary that includes (as applicable): background, objectives, eligibility criteria, sources of evidence, charting methods, results, and conclusions that relate to the review questions and objectives.	
**INTRODUCTION**			
Rationale	3	Describe the rationale for the review in the context of what is already known. Explain why the review questions/objectives lend themselves to a scoping review approach.	
Objectives	4	Provide an explicit statement of the questions and objectives being addressed with reference to their key elements (e.g., population or participants, concepts, and context) or other relevant key elements used to conceptualize the review questions and/or objectives.	
**METHODS**			
Protocol and registration	5	Indicate whether a review protocol exists; state if and where it can be accessed (e.g., a Web address); and if available, provide registration information, including the registration number.	
Eligibility criteria	6	Specify characteristics of the sources of evidence used as eligibility criteria (e.g., years considered, language, and publication status), and provide a rationale.	
Information sources*	7	Describe all information sources in the search (e.g., databases with dates of coverage and contact with authors to identify additional sources), as well as the date the most recent search was executed.	
Search	8	Present the full electronic search strategy for at least 1 database, including any limits used, such that it could be repeated.	
Selection of sources of evidence†	9	State the process for selecting sources of evidence (i.e., screening and eligibility) included in the scoping review.	
Data charting process‡	10	Describe the methods of charting data from the included sources of evidence (e.g., calibrated forms or forms that have been tested by the team before their use, and whether data charting was done independently or in duplicate) and any processes for obtaining and confirming data from investigators.	
Data items	11	List and define all variables for which data were sought and any assumptions and simplifications made.	
Critical appraisal of individual sources of evidence§	12	If done, provide a rationale for conducting a critical appraisal of included sources of evidence; describe the methods used and how this information was used in any data synthesis (if appropriate).	
Synthesis of results	13	Describe the methods of handling and summarizing the data that were charted.	

SECTION	ITEM	PRISMA-ScR CHECKLIST ITEM	REPORTED ON PAGE #
**RESULTS**			
Selection of sources of evidence	14	Give numbers of sources of evidence screened, assessed for eligibility, and included in the review, with reasons for exclusions at each stage, ideally using a flow diagram.	
Characteristics of sources of evidence	15	For each source of evidence, present characteristics for which data were charted and provide the citations.	
Critical appraisal within sources of evidence	16	If done, present data on critical appraisal of included sources of evidence (see item 12).	
Results of individual sources of evidence	17	For each included source of evidence, present the relevant data that were charted that relate to the review questions and objectives.	
Synthesis of results	18	Summarize and/or present the charting results as they relate to the review questions and objectives.	
**DISCUSSION**			
Summary of evidence	19	Summarize the main results (including an overview of concepts, themes, and types of evidence available), link to the review questions and objectives, and consider the relevance to key groups.	
Limitations	20	Discuss the limitations of the scoping review process.	
Conclusions	21	Provide a general interpretation of the results with respect to the review questions and objectives, as well as potential implications and/or next steps.	
**FUNDING**			
Funding	22	Describe sources of funding for the included sources of evidence, as well as sources of funding for the scoping review. Describe the role of the funders of the scoping review.	

JBI = Joanna Briggs Institute; PRISMA-ScR = Preferred Reporting Items for Systematic reviews and Meta-Analyses extension for Scoping Reviews.

* Where *sources of evidence* (see second footnote) are compiled from, such as bibliographic databases, social media platforms, and Web sites.

† A more inclusive/heterogeneous term used to account for the different types of evidence or data sources (e.g., quantitative and/or qualitative research, expert opinion, and policy documents) that may be eligible in a scoping review as opposed to only studies. This is not to be confused with *information sources* (see first footnote).

‡ The frameworks by Arksey and O'Malley (6) and Levac and colleagues (7) and the JBI guidance (4, 5) refer to the process of data extraction in a scoping review as data charting.

§ The process of systematically examining research evidence to assess its validity, results, and relevance before using it to inform a decision. This term is used for items 12 and 19 instead of "risk of bias" (which is more applicable to systematic reviews of interventions) to include and acknowledge the various sources of evidence that may be used in a scoping review (e.g., quantitative and/or qualitative research, expert opinion, and policy document).

From: Tricco AC, Lillie E, Zarin W, O'Brien KK, Colquhoun H, Levac D, et al. PRISMA Extension for Scoping Reviews (PRISMAScR): Checklist and Explanation. Ann Intern Med. 2018;169:467–473. doi: 10.7326/M18-0850.

Search Strategy

Search interface: PubMed Advanced Search Builder

Initial search; date run- 11 September 2024
Second search; date run- 22 March 2026

(network*[tiab] OR pathway*[tiab] OR connect*[tiab] OR circuit*[tiab] OR "functional connectivity"[tiab] OR "brain connectivity"[tiab]) AND (therap*[tiab] OR treat*[tiab] OR interven*[tiab] OR experiment*[tiab] OR stimulat*[tiab] OR neurostimulat*[tiab] OR neuromodulat*[tiab] OR "deep brain stimulation"[tiab] OR tDCS[tiab] OR TMS[tiab]) AND ("minimally conscious state"[tiab] OR "minimally conscious"[tiab] OR "minimally responsive state"[tiab] OR "vegetative state"[tiab] OR "persistent vegetative state"[tiab] OR "unresponsive wakefulness syndrome"[tiab] OR "prolonged disorders of consciousness"[tiab] OR "chronic coma"[tiab] OR "prolonged coma"[tiab])

Limits:
None applied during initial search.

Supplementary Table S1

Study	DoC cohort (N)	Study design	Modality	Anatomical target	Putative network/system	Protocol	Group-level clinical outcome	Neurophysiological / neuroimaging assessment
Bai et al. 2018	MCS (8); UWS (9)	Sham-controlled crossover	tDCS	Left DLPFC (F3)	FPN	2 mA; 20 min	No significant improvement	EEG coherence
Barra et al. 2022	MCS (10); eMCS (2)	Randomized, double-blind, sham-controlled crossover pilot	tDCS / tPCS	Left prefrontal cortex (F3); bilateral mastoids	FPN	20 min	No significant primary effect	EEG dwPLI
Carrière et al. 2020	MCS- (6); MCS+ (4); eMCS (1)	Randomized, double-blind, sham-controlled crossover pilot	tDCS	Left DLPFC (F3)	FPN	2 mA; 20 min	No significant improvement	High-density EEG; wSMI/wPLI
Cavinato et al. 2019	MCS (12); UWS (12)	Randomized, double-blind, sham-controlled crossover	tDCS	Left DLPFC (F3)	FPN	Repeated 2 mA; 20 min	No significant CRS-R improvement	EEG coherence
Dang et al. 2023	MCS (9)	Sham-controlled crossover with open chronic phase	DBS	Bilateral CM-Pf nuclei	Central thalamic / thalamocortical	Bilateral 100 Hz DBS	No significant controlled group effect	EEG connectivity and graph analysis
He et al. 2018	VS (3); MCS (2); eMCS (1)	Randomized, sham-controlled crossover	rTMS	Left M1	Sensorimotor / pericentral	20 Hz; 1,000 pulses; 5 days	No significant improvement	EEG spectral power
Huang et al. 2024	VS/UWS (18)	Randomized, double-blind, sham-controlled crossover	iTBS	Left DLPFC	FPN	600 pulses; 80% RMT; single session	No significant improvement	EEG wPLI
Lemaire	MCS- (4); UWS	Prospective	DBS	Central-anterior	Motor Network	Chronic DBS	No significant	FDG-PET

et al. 2018	(1)	pilot with blinded ON/OFF phase		thalamus + medial pallidum			controlled group effect	
Li et al. 2024	MCS (20)	Randomized, sham-controlled	rTMS	Pz / precuneus	DMN	10 Hz; 1,000 pulses; 90% RMT; 10 sessions	Significant improvement	EEG microstates
Li et al. 2025	MCS (20)	Randomized, sham-controlled	rTMS	Left DLPFC (F3) + precuneus (Pz)	FPN + DMN (cross-network)	10 Hz; 1,000 pulses; 90% RMT; 10 sessions	Significant improvement	EEG coherence
Liu et al. 2018	MCS (5); UWS/VS (2)	Randomized, double-blind, sham-controlled crossover	rTMS	Left M1	Sensorimotor / pericentral	20 Hz; 5 days	No significant improvement	Resting-state fMRI
Mancuso et al. 2017	VS/UWS (9)	Randomized, double-blind, sham-controlled pilot	tRNS	Bilateral DLPFC (F3/F4)	FPN	2 mA; 20 min/day; 5 days	No significant improvement	Semiquantitative EEG
Martens et al. 2020	UWS (17); MCS (23); eMCS (6)	Double-blind, sham-controlled crossover RCT	Multifocal tDCS	F3, F4, CP5, CP6	Bilateral FPN-associated multisite	1 mA/electrode; 20 min	No significant improvement	EEG power and LZW complexity
Naro et al. 2015	Post-anoxic UWS (10)	Prospective pilot; sham tested in responders	rTMS	Right DLPFC (F4)	FPN	10 Hz; 1,000 pulses; 90% RMT; single session	No significant improvement	Paired/dual-coil TMS
Naro et al. 2016	MCS (12); UWS (14)	Randomized-order active-control study	tACS / tRNS	Right DLPFC (F4)	FPN	Gamma-range tACS; active-control conditions	No significant improvement	EEG partial directed coherence
Thibaut et al. 2014	MCS (30); VS/UWS (25)	Randomized, double-blind, sham-	tDCS	Left DLPFC (F3)	FPN	2 mA; 20 min; single session	Significant improvement in MCS; not	None

		controlled crossover					VS/UWS	
Thibaut et al. 2015	MCS (21)	Retrospective, secondary responder analysis	tDCS	Left DLPFC (F3)	FPN	2 mA; 20 min	Secondary analysis; not an independent efficacy trial	Structural MRI; FDG-PET; EEG
Xia et al. 2019	MCS (7); VS (14)	Prospective physiological pre/post study	rTMS	Left DLPFC	FPN	10 Hz; 1,000 pulses; 90% RMT; single session	Clinical treatment effect not assessed	TMS-EEG
Xu et al. 2023	VS/UWS (20)	Randomized, double-blind, sham-controlled crossover	rTMS	Left PPC (P3)	DMN-associated*	10 Hz; 2,000 pulses/day; 90% RMT; 10 sessions	Significant improvement	EEG spectral power
Yan et al.2026	MCS (24)	Randomized, double-blind, sham-controlled	rTMS	Pz / precuneus	DMN	10 Hz; 90% RMT; 10 days	Significant improvement	EEG phase-locking value
Zhou et al. 2023	MCS (50)	Randomized, double-blind, sham-controlled	taVNS	Left cymba conchae	Salience network (putative indirect modulation)†	20 Hz; 30 min twice daily; 4 weeks	Significant improvement	EEG; USEP; BAEP; P300
Zhang et al. 2020	MCS (5), UWS (8)	Prospective open-label interventional study (all active tDCS)	tDCS	Left DLPFC	FPN	2 mA; 20 min; 20 anodal sessions over 10 days	Within group pre-/post-significant improvement. Not control group led effect; classed as non-responder	ERPs (P300); FDG-PET

Supplementary Table S1: Study-level characteristics for each study included in the scoping review. DoC cohort, Study Design, Stimulation Modality, Anatomical Target, Putative Network/ System, Protocol, Group-level Clinical Outcome and Neurophysiological/ Neuroimaging assessment utility specified.
